# Tarlatamab in small cell lung cancer following platinum-based chemotherapy: subgroup analysis of Japanese patients from the DeLLphi-304 study

**DOI:** 10.1007/s10147-026-03192-y

**Published:** 2026-09-19

**Authors:** Takayasu Kurata, Shunichi Sugawara, Hiroaki Akamatsu, Kazushige Wakuda, Koichi Azuma, Takayuki Takahama, Hiroshi Kagamu, Hiroshi Yokouchi, Miyako Satouchi, Shuji Murakami, Kadoaki Ohashi, Makoto Nishio, Kazumi Nishino, Hiroki Izumi, Hiroshi Tanaka, Taichi Miyawaki, Shuang Huang, Upen Patil, Jihyun Park, Tatsuya Yoshida

**Affiliations:** 1 Department of Thoracic Oncology, Kansai Medical University Hospital, 2-3-1 Shin-machi, Hirakata city, Osaka 573-1191 Japan; 2 Sendai Kousei Hospital, Miyagi, Japan; 3 Internal Medicine III, Wakayama Medical University Hospital, Wakayama, Japan; 4 Division of Thoracic Oncology, Shizuoka Cancer Center, Shizuoka, Japan; 5 Division of Respirology, Neurology, and Rheumatology, Department of Internal Medicine, Kurume University School of Medicine, Fukuoka, Japan; 6 Department of Medical Oncology, Kindai University Faculty of Medicine, Osaka-Sayama, Japan; 7 Department of Respiratory Medicine, International Medical Center, Saitama Medical University, Saitama, Japan; 8 Department of Respiratory Medicine, NHO Hokkaido Cancer Center, Sapporo, Japan; 9 Department of Thoracic Oncology, Hyogo Cancer Center, Akashi, Japan; 10 Department of Thoracic Oncology, Kanagawa Prefectural Hospital Organization Kanagawa Cancer Center, Yokohama, Japan; 11 Department of Respiratory Medicine, Okayama University Hospital, Okayama, Japan; 12 Department of Thoracic Medical Oncology, The Cancer Institute Hospital of Japanese Foundation for Cancer Research, Tokyo, Japan; 13 Department of Thoracic Oncology, Osaka International Cancer Institute, Osaka, Japan; 14 Department of Thoracic Oncology, National Cancer Center Hospital East, Chiba, Japan; 15 Department of Internal Medicine, Niigata Cancer Center Hospital, Niigata, Japan; 16 Department of Respiratory Medicine, Graduate School of Medicine, Juntendo University, Tokyo, Japan; 17 Amgen Inc, Thousand Oaks, CA USA; 18 Amgen K.K, Tokyo, Japan; 19 Department of Thoracic Oncology, National Cancer Center Hospital, Tokyo, Japan

**Keywords:** DeLLphi-304, Japanese patients, Small-cell lung cancer, Tarlatamab

## Abstract

**Background:**

In the Phase 3 DeLLphi-304 trial, tarlatamab significantly improved overall survival (OS) over chemotherapy for small-cell lung cancer (SCLC) progressed after platinum-based chemotherapy. We report efficacy and safety in the Japanese subpopulation.

**Methods:**

Patients were randomized to tarlatamab or amrubicin in Japan. The primary endpoint was OS. Secondary endpoints included progression-free survival (PFS), objective response rate (ORR), duration of response (DOR), and safety.

**Results:**

As of 29 Jan 2025 (data cutoff), 35 patients (tarlatamab, *n* = 12; amrubicin, *n* = 23) were enrolled. Baseline characteristics were generally balanced although fewer tarlatamab-treated patients had chemotherapy-free interval < 90 days or brain metastases. Median OS was NE (95% CI, 10.4, NE) for tarlatamab versus 11.5 (6.2, NE) months for amrubicin (HR [95% CI] = 0.533 [0.186, 1.529]). Twelve-month survival was 66.7% (33.7, 86.0) versus 47.0% (25.7, 65.6), respectively. Median PFS was 4.0 (1.3, 11.3) months versus 4.2 (2.1, 5.4) months (HR [95% CI] = 0.726 [0.339, 1.554]). ORR was 33.3% (9.9, 65.1) versus 26.1% (10.2, 48.4). Median DOR was 10.1 (3.0, NE) months versus 5.6 (2.8, NE) months. Grade ≥ 3 treatment-related adverse events were less frequent with tarlatamab (8.3%) versus amrubicin (56.5%). Cytokine release syndrome occurred in 75% of tarlatamab-treated patients (all were grade 1 or 2) and grade 2 immune effector cell-associated neurotoxicity syndrome was reported in 1 (8.3%) patient.

**Conclusion:**

Tarlatamab demonstrated numerically longer OS and DOR, and higher ORR versus amrubicin in Japanese patients with SCLC, with fewer high-grade treatment-related adverse events. These results in Japanese patients support those of the global population.

**Supplementary Information:**

The online version contains supplementary material available at https://doi.org/10.1007/s10147-026-03192-y.

## Introduction

Small cell lung cancer (SCLC) has a poor prognosis with a 5-year relative survival of less than 3% reported in Japanese patients with extensive-stage SCLC, and most patients experience relapse within 6 months after achieving a complete response with first-line treatment [1–5].

International clinical practice guidelines recommend tarlatamab, irinotecan, lurbinectedin, topotecan, nivolumab, pembrolizumab and other single-agent therapies as second-line therapy [5, 6]. Amrubicin has demonstrated efficacy in Japanese patients with both platinum-sensitive and -refractory SCLC [7–9], and is recommended as a second-line treatment option in Japanese clinical guidelines.

Tarlatamab, a bispecific T-cell engager immunotherapy, targets the Notch signaling delta-like ligand 3 (DLL3) expressed on the surface of cancer cells in up to 96% of patients with SCLC as well as CD3 on T cells, resulting in cancer-cell lysis independent of major histocompatibility complex class I recognition [10–13]. In the phase 2, open-label, global DeLLphi-301 trial, tarlatamab demonstrated clinically meaningful anticancer activity characterized by durable objective responses and favourable survival outcomes in patients with previously treated SCLC [10]. Based on these positive results, tarlatamab received approval in Japan in late 2024 for the treatment of previously treated SCLC [14]. In the multinational, open-label, randomized controlled DeLLphi-304 trial, 509 patients with SCLC who had experienced disease progression during or after initial platinum-based therapy were randomly assigned to receive tarlatamab (*n* = 254) or chemotherapy (*n* = 255) [15]. Tarlatamab significantly improved overall survival (OS) compared with chemotherapy (stratified hazard ratio [HR] for death, 0.60; 95% CI 0.47 to 0.77; *P* < 0.001) and significantly prolonged progression-free survival (PFS; piecewise weighted average HR for progression or death, 0.71; 95% CI 0.59 to 0.86; *P* = 0.002). In addition, tarlatamab demonstrated a favorable safety profile, with a lower incidence of Grade ≥ 3 adverse events (AEs) compared with chemotherapy.

Differences in healthcare systems and patient demographics can influence disease course and clinical outcomes [16, 17]. Accordingly, regional treatment guidelines have been developed to account for potential geographic and ethnic variations that may lead to differences in clinical management [6, 18]. Several studies have reported that, despite the prognosis of SCLC being universally poor, there are differences in OS and toxicity by ethnicity, particularly between East Asian and European/North American populations [19, 20]. These potential ethnic variations underscore the need to evaluate the efficacy of therapies in Japanese patients to inform appropriate clinical treatment decision making.

Here, we report the results from the DeLLphi-304 trial (NCT05740566) of tarlatamab versus chemotherapy in Japanese patients with SCLC who experienced disease progression during or after first-line platinum-based chemotherapy.

## Patients and methods

### Study design

The study design of DeLLphi-304 has been described in detail previously [15]. In brief, DeLLphi-304 was a multicenter, randomized, controlled Phase 3 study in patients in whom SCLC had progressed after first-line platinum-based chemotherapy ± anti-PD-(L)1.

The trial was conducted in accordance with the protocol and international guidelines, including the Declaration of Helsinki, and all applicable laws and regulations. The protocol and amendments were approved by the appropriate ethics body at each participating center.

### Patients

The study design and eligibility criteria for DeLLphi-304 have been previously described [15]. Briefly, patients of age 18 years or older with SCLC that had progressed during or after first-line platinum-based chemotherapy, and ECOG PS 0 or 1 were eligible. Patients with asymptomatic and clinically stable brain metastases were permitted, regardless of treatment status, and DLL3 expression was not required for trial participation. (see Supplementary Table 1 for full inclusion and exclusion criteria). Eligible patients in the overall study population were randomized 1:1 to receive tarlatamab or amrubicin and stratified by prior anti-PD-(L)1 exposure, chemotherapy-free interval, brain metastases, and intended chemotherapy.

### Treatments

Tarlatamab was administered as a 60-minute intravenous infusion with a step-up dose of 1 mg on day 1 of cycle 1, followed by 10 mg on days 8 and 15 of cycle 1 and then 10 mg every 2 weeks thereafter in 28-day cycles. For chemotherapy, Japanese patients received amrubicin administered intravenously at a dose of 40 mg/m^2^ on days 1 through 3 every 3 weeks. Trial treatment continued until the occurrence of investigator-determined radiologic disease progression according to the Response Evaluation Criteria in Solid Tumors (RECIST), 1.1, unacceptable toxicity, withdrawal of consent, or death. Continuation of the trial treatment after the occurrence of disease progression was allowed on the basis of investigator determination of clinical benefit.

### Endpoints

The primary endpoint was OS, defined as the time from randomization to death from any cause. Key secondary endpoints included investigator-assessed PFS, defined as the time from randomization to the disease progression according to RECIST 1.1, or death from any cause, whichever occurred first, as well as patient-reported outcomes (PROs). PROs were not analyzed in the Japanese subpopulation analysis. Objective response rate (ORR), disease control, and duration of response (DOR), as assessed by investigators per RECIST 1.1, and safety were also evaluated as a secondary endpoint.

### Statistical analysis

Analyses of efficacy and safety data for tarlatamab versus chemotherapy in Japanese patients were exploratory and were summarized using descriptive statistics. The statistical analysis, including sample size calculation for the overall population, has been described in detail in the original DeLLphi-304 trial report [15]. Efficacy analyses were conducted in all randomized Japanese patients. OS and PFS were estimated using the Kaplan-Meier method; HRs and the corresponding 95% CI were estimated using an unstratified Cox proportional hazards model for the Japanese subpopulation analysis. For DOR, median and percentiles were estimated using the Kaplan-Meier method with 95% CI estimated using log-log transformation by the Brookmeyer and Crowley method [21].

SAS^®^ version 9.4 (SAS Institute Inc., Cary, NC, USA) was used for all statistical analyses.

## Results

### Patients and treatment

Among Japanese patients, 12 patients were randomized to receive tarlatamab and 23 patients to amrubicin. Baseline characteristics were generally balanced between the tarlatamab and amrubicin groups (Table 1). However, a lower proportion of patients in the tarlatamab versus amrubicin groups had never smoked (0% versus 13%), chemotherapy-free interval < 90 days (33.3% versus 52.2%), and presence of brain metastases (33.3% versus 52.2%). The median age of the patients was 67 years (range 49 to 76) in the tarlatamab group and 70 years (range 26 to 84) in the amrubicin group. Liver metastases were present in 41.7% and 43.5% of patients in tarlatamab and amrubicin groups, respectively. Prior treatment with PD-L1 or PD-1 inhibitor was reported in 91.7% of patients in the tarlatamab group and 87.0% in the amrubicin group. Platinum-resistant disease, defined as disease progression after a chemotherapy-free interval of less than 90 days after the final dose of first-line platinum-based chemotherapy, was observed in 33.3% of patients in tarlatamab group and 52.2% in the amrubicin group. As of the data-cutoff date (January 29, 2025), the median duration of treatment was 16.0 weeks in the tarlatamab group and 11.6 weeks in the amrubicin group.

**Table 1:** Baseline demographic and clinical characteristics of Japanese patients enrolled in the DeLLphi-304 trial

	Tarlatamab (*n* = 12)	Amrubicin (*n* = 23)
Median age, years (range)	67 (49–76)	70 (26–84)
Male/Female, n (%)	9 (75)/3 (25)	17 (74)/6 (26)
ECOG performance status, n (%) 0 1 2	4 (33.3) 8 (66.7) 0 (0.0)	9 (39.1) 14 (60.9) 0 (0.0)
Smoking history, n (%) Never Current Former	0 (0.0) 2 (16.7) 10 (83.3)	3 (13.0) 1 (4.3) 19 (82.6)
Prior anti-PD-(L)1 therapy, n (%)	11 (91.7)	20 (87.0)
Prior radiotherapy for current malignancy, n (%)	5 (41.7)	9 (39.1)
Chemotherapy-free interval, n (%) < 90 days ≥ 90 to < 180 days ≥ 180 days	4 (33.3) 4 (33.3) 4 (33.3)	12 (52.2) 3 (13.0) 8 (34.8)
Presence of brain metastases, n (%)	4 (33.3)	12 (52.2)
Presence of liver metastases, n (%)	5 (41.7)	10 (43.5)
DLL3 expression, n/N (%)^a^	6/9 (66.7)	17/20 (85.0)

Note: ^a^Percentage for DLL3 expression is based on subjects with available tissue sample that could be evaluated for DLL3 expression as the denominator (N)

### Efficacy

**Fig. 1:**
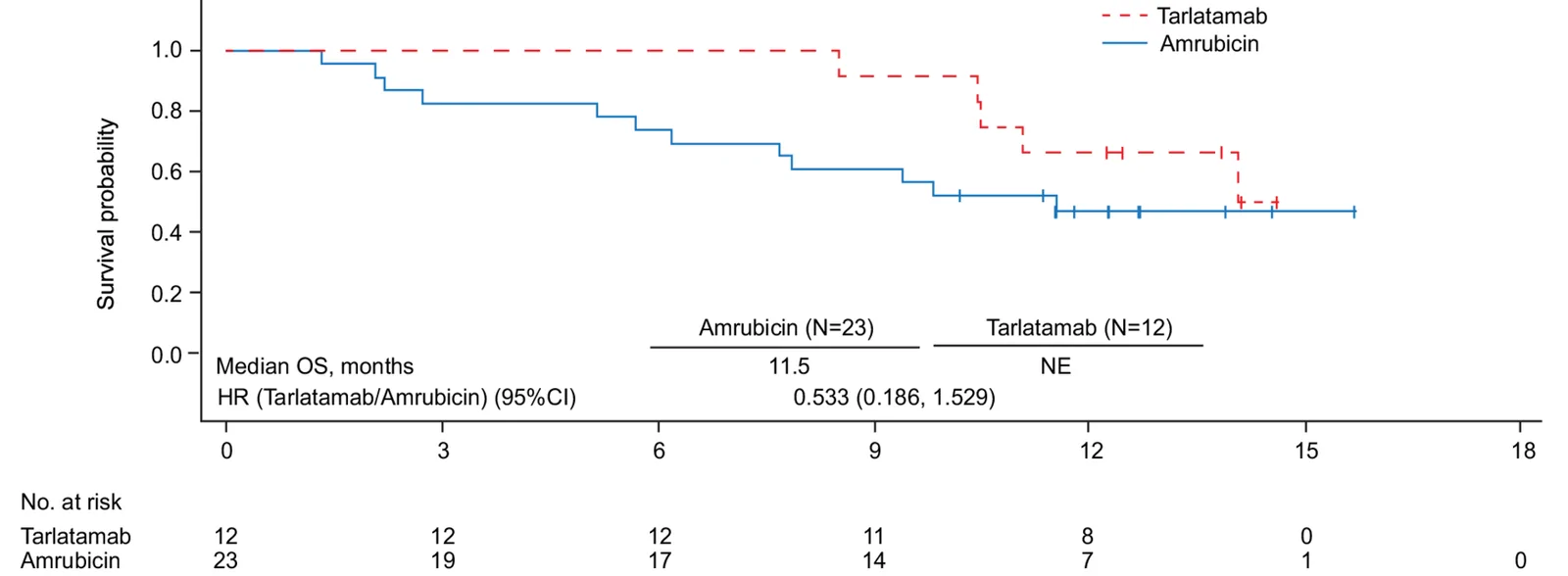
Overall survival in Japanese patients enrolled in the DeLLphi-304 trial

**Fig. 2:**
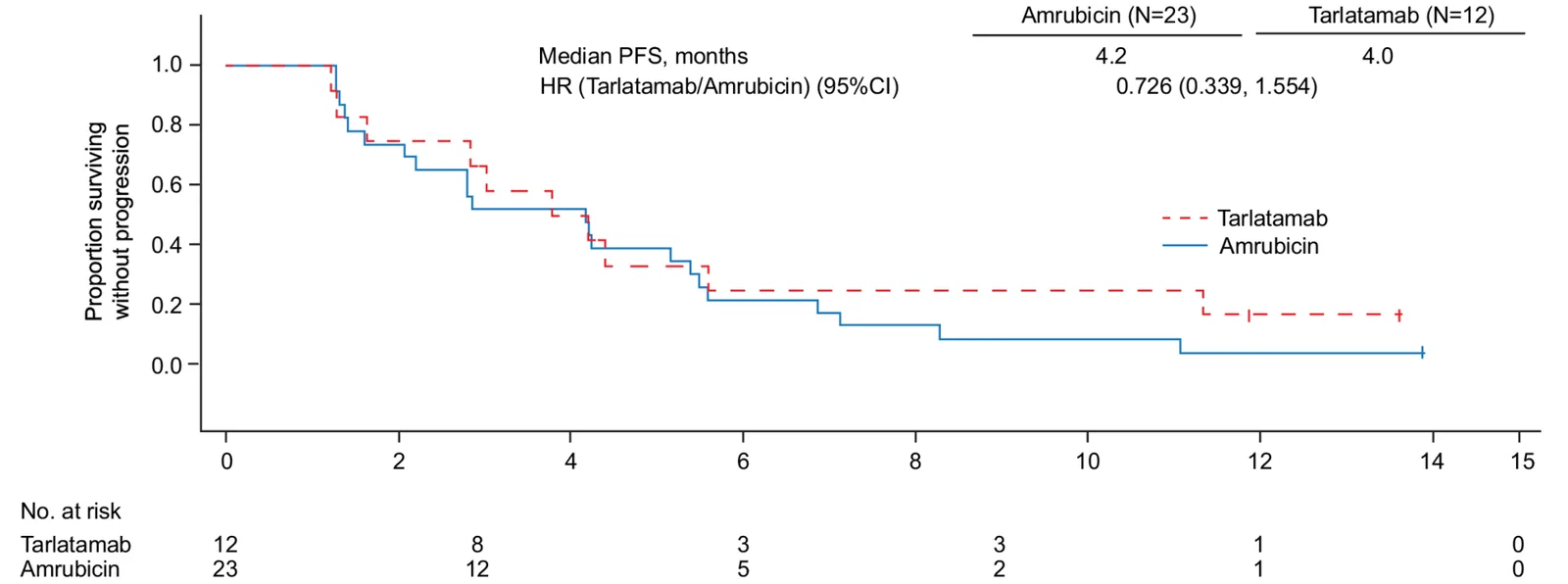
Progression-free survival in Japanese patients enrolled in the DeLLphi-304 trial

As of the data-cutoff date, the median duration of follow-up for OS was 14.0 months in the tarlatamab group and 12.3 months in the amrubicin group. Among all randomized Japanese patients, median OS was not reached (95% CI, 10.4, not estimable [NE]) for tarlatamab versus 11.5 months (95% CI 6.2, NE) for amrubicin (HR [95% CI] = 0.533 [0.186, 1.529]) (Fig. 1); Kaplan-Meier estimates of 12-month OS rates were 66.7% (95% CI 33.7, 86.0) for tarlatamab versus 47.0% (95% CI 25.7, 65.6) for amrubicin. Median PFS was 4.0 months (95% CI 1.3, 11.3) for tarlatamab versus 4.2 months (95% CI 2.1, 5.4) for amrubicin (HR [95% CI] = 0.726 [0.339, 1.554]) (Fig. 2). The Kaplan-Meier estimates of PFS were 25.0% at 6 months and 16.7% at 12 months in the tarlatamab group as compared with 21.7% at 6 months and 4.3% at 12 months in the amrubicin group. ORR (95% CI) was 33.3% (9.9, 65.1) for tarlatamab versus 26.1% (10.2, 48.4) for amrubicin (odds ratio [95% CI] = 1.42 [0.31, 6.47]) (Table 2). The median DOR was 10.1 months (95% CI, 3.0, NE) in the tarlatamab group and 5.6 months (95% CI, 2.8, NE) in the amrubicin group. The Kaplan-Meier estimate of the 12-month duration of response was 37.5% (95% CI 1.1, 80.8) and 16.7% (95% CI 0.8, 51.7) for tarlatamab and amrubicin, respectively (Table 3). In terms of best overall response, no complete responses were observed. The confirmed partial response (PR) rate was 33.3% (4 out of 12) in the tarlatamab group and 26.1% (6 out of 23) in the amrubicin group. The disease control rate (DCR) was 75.0% in the tarlatamab group and 69.6% in the amrubicin group. After the occurrence of disease progression, subsequent anti-cancer therapy was received by 10 of 12 patients (83.3%) in the tarlatamab arm and 15 of 23 patients (65.2%) in the amrubicin arm.

**Table 2:** Summary of objective response in Japanese patients enrolled in the DeLLphi-304 trial as assessed by investigator

	Tarlatamab (*n* = 12)	Amrubicin (*n* = 23)
Best overall response^a^, *n* (%)		
Confirmed complete response	0 (0.0)	0 (0.0)
Confirmed partial response	4 (33.3)	6 (26.1)
Stable disease	5 (41.7)	10 (43.5)
Progressive disease	3 (25.0)	5 (21.7)
Not evaluable	0 (0.0)	0 (0.0)
No post-baseline scan	0 (0.0)	2 (8.7)
ORR per investigator, % (95% CI)^b^	33.3 (9.9, 65.1)	26.1 (10.2, 48.4)
Odds ratio (95% CI)^c^	1.42 (0.31, 6.47)	
DCR, % (95% CI)^b^	75.0 (42.8, 94.5)	69.6 (47.1, 86.8)

Abbreviations: CI, confidence interval; DCR, disease control rate; ORR, objective response rate

Note: ^a^Determined using RECIST 1.1 guidelines; ^b^Exact confidence interval calculated using Clopper Pearson method; ^c^Odds ratio with 95% CI estimated using Cochran Mantel Haenszel Chi-square test

**Table 3:** Summary of duration of response in Japanese patients enrolled in the DeLLphi-304 trial as assessed by investigator

	Tarlatamab (*n* = 12)	Amrubicin (*n* = 23)
Number of confirmed responders	4	6
Events^a^, n (%)	2 (50.0)	5 (83.3)
Censored^b^, n (%)	2 (50.0)	1 (16.7)
Duration of response (KM), months		
25th percentile (95% CI)	6.5 (3.0, NE)	4.2 (2.8, 6.9)
Median (95% CI)	10.1 (3.0, NE)	5.6 (2.8, NE)
75th percentile (95% CI)	NE (3.0, NE)	8.5 (4.2, NE)
KM estimate (%) (95% CI)		
At 3 months	75.0 (12.8, 96.1)	83.3 (27.3, 97.5)
At 6 months	75.0 (12.8, 96.1)	50.0 (11.1, 80.4)
At 9 months	75.0 (12.8, 96.1)	16.7 (0.8, 51.7)
At 12 months	37.5 (1.1, 80.8)	16.7 (0.8, 51.7)

Abbreviations: CI, confidence interval; KM, Kaplan-Meier

Note: ^a^Disease progression in all cases; ^b^On study without disease progression or death in all cases

### Safety

Among treated patients, any-cause AEs were observed in all patients in both the tarlatamab (12 patients, 100%) and amrubicin (23 patients, 100%) groups (Table 4). AEs of Grade ≥ 3 occurred in 66.7% of tarlatamab-treated patients and 73.9% of amrubicin-treated patients. Any-Grade treatment-related AEs (TRAEs) occurred in 12 (100%) patients in tarlatamab group and 21 (91.3%) patients in amrubicin group. The incidence of Grade ≥ 3 TRAEs was numerically lower with tarlatamab (*n* = 1, 8.3%) versus amrubicin (*n* = 13, 56.5%) (Supplementary Table 2, Supplementary Table 3). The most common (≥ 10%) TRAEs reported were cytokine release syndrome (CRS; 75.0%) and dysgeusia (66.7%) in the tarlatamab group and anemia (52.2%), neutropenia (34.8%), and febrile neutropenia (30.4%) in the amrubicin group. Treatment-related serious adverse events were reported in none of the patients in the tarlatamab group and in 34.8% of those in the amrubicin group. Any grade TRAEs leading to dose interruption and/or dose reduction were reported in 16.7% of patients in the tarlatamab group and 56.5% in the amrubicin group. No TRAEs led to treatment discontinuation in either treatment groups. Treatment-related Grade 5 events were observed in no patients in the tarlatamab group and 1 patient in the amrubicin group (pneumonia).

**Table 4:** Summary of treatment-emergent adverse events and treatment-related adverse events in Japanese patients enrolled in the DeLLphi-304 trial

	Tarlatamab (*n* = 12)	Amrubicin (*n* = 23)
All grade TEAEs, *n* (%)	12 (100.0)	23 (100.0)
All grade TRAEs, n (%)	12 (100.0)	21 (91.3)
Grade ≥ 3 TRAEs, n (%)	1 (8.3)	13 (56.5)
Serious TRAEs, n (%)	0 (0.0)	8 (34.8)
TRAEs leading to dose interruption and/or dose reduction, n (%)	2 (16.7)	13 (56.5)
TRAEs leading to discontinuation, n (%)	0 (0.0)	0 (0.0)
Treatment-related Grade 5 events^a^, n (%)	0 (0.0)	1 (4.3)
TRAEs reported in ≥ 10% of patients, n (%)		
Cytokine release syndrome	9 (75.0)	0 (0.0)
Dysgeusia	8 (66.7)	1 (4.3)
Anemia	2 (16.7)	12 (52.2)
Neutropenia	2 (16.7)	8 (34.8)
Constipation	2 (16.7)	6 (26.1)
Leukopenia	1 (8.3)	6 (26.1)
Decreased appetite	1 (8.3)	4 (17.4)
Stomatitis	1 (8.3)	3 (13.0)
Febrile neutropenia	0 (0.0)	7 (30.4)
Malaise	0 (0.0)	6 (26.1)
Thrombocytopenia	0 (0.0)	5 (21.7)
Nausea	0 (0.0)	4 (17.4)
Platelet count decreased	0 (0.0)	3 (13.0)

Abbreviations: TEAE, treatment-emergent adverse event; TRAE, treatment-related adverse event

^a^Related to pneumonia

Note: Treatment-emergent adverse events (TEAEs) were adverse events that occurred or worsened after initiation of study treatment, regardless of causality. Treatment-related adverse events (TRAEs) were TEAEs considered by the investigator to have a reasonable possibility of being related to study treatment.

CRS and immune effector cell-associated neurotoxicity syndrome (ICANS) were anticipated adverse events based on the mechanism of action of tarlatamab, while dysgeusia was assessed due to its prior reporting in clinical trials. CRS occurred in 75% of patients (9 out of 12) in the tarlatamab group; events were Grade 1 in 8 patients and Grade 2 in 1 patient, with no Grade 3 or higher events reported (Table 5). CRS occurred primarily after the receipt of one of the first two doses of tarlatamab. The median time to onset of CRS events was 25.0 h (IQR 12.7–38.5) following the first dose of tarlatamab. Corticosteroids were administered for the management of CRS in 2 (16.7%) patients, IV fluids in 1 (8.3%) patient, and low-flow (≤ 6 L/min) supplemental oxygen in 1 (8.3%) patient (Table 5). The median time to CRS intervention following the most recent tarlatamab was 5.5 days, with a range from 2 to 16 days. All cases of CRS resolved, with a median time to resolution of 2 days (IQR 2.0–3.0). Treatment-related ICANS was reported in 1 (8.3%) patient treated with tarlatamab (Grade 2). The time to onset for ICANS was 8 days in this patient. Treatment-related dysgeusia was observed in 8 (66.7%) patients treated with tarlatamab (Grade 1, *n* = 6; Grade 2, *n* = 2) and 1 patient treated with amrubicin (Grade 1). In the tarlatamab arm, the median time to onset was 25.0 days (IQR 17.5–36.5) in the tarlatamab group and 10.0 days (IQR 10.0–10.0) in the amrubicin group. Resolution of dysgeusia was observed in 1 patient, with a mean duration of 131.0 days. Unresolved events were defined as events with an unobserved stop date, either because the event was ongoing at the time of data cutoff, the stop date was missing, follow-up ended because of death, or the event extended beyond the adverse event assessment period.

**Table 5:** Summary of cytokine release syndrome events and interventions

	Tarlatamab (*n* = 12)
All treatment-emergent CRS events, *n* (%)	9 (75.0)
Grade 1 events	8 (66.7)
Grade 2 events	1 (8.3)
Grade ≥ 3 events	0 (0)
Utilization of CRS interventions, n (%)	3 (25.0)
Tocilizumab	0 (0)
Corticosteroids	2 (16.7)
Vasopressor	0 (0)
IV fluids	1 (8.3)
Supplemental oxygen	1 (8.3)
Low-flow (≤ 6 L/min) oxygen	1 (8.3)
High-flow (> 6 L/min) oxygen	0 (0)

## Discussion

In this Japanese subpopulation of DeLLphi-304 trial, efficacy outcomes were consistent with the findings in the primary analysis. Tarlatamab demonstrated a clinically meaningful improvement in OS versus amrubicin (HR [95% CI] = 0.533 [0.186, 1.529]), with generally comparable PFS between treatment arms. Although median PFS was similar between treatment arms, the 12-month PFS rate was numerically higher with tarlatamab than with amrubicin. However, this observation should be interpreted cautiously because of the exploratory nature of the analysis, the small sample size, and the very limited number of patients remaining at risk at 12 months. Other efficacy outcomes, including ORR and DOR also favoured tarlatamab over amrubicin. These findings in the Japanese subpopulation were consistent with those observed in the overall randomized population of this global study [15]. No new safety signals were observed among tarlatamab-treated patients in the Japanese subpopulation, with a lower incidence of Grade 3 or higher TRAEs compared with amrubicin. Although interpretation is limited due to the small sample size, there was a numerically higher incidence of dysgeusia and CRS observed in Japanese patients treated with tarlatamab compared with the global tarlatamab-treated population. However, no Grade ≥ 3 CRS events were reported; cases were predominantly low grade (Grade 1 in 8 patients and Grade 2 in 1 patient) and did not lead to treatment interruption or discontinuation. Further investigation is needed to better characterize the incidence of dysgeusia in Japanese patients. ICANS, another recognized AE associated with tarlatamab, occurred in one patient and was of low grade (Grade 2); the event subsequently resolved. In this clinical trial, most CRS and ICANS events were Grade 1 or 2 and were generally manageable. In real-world practice, however, a broader patient population may be treated with tarlatamab, and appropriate management of AEs such as CRS and ICANS will be crucial. Careful symptom monitoring, along with cautious and proactive management, should be maintained throughout tarlatamab treatment.

Immunotherapy, including immune checkpoint inhibitors such as anti-PD-(L)1 inhibitors, has become an important component of first-line treatment for several cancer types, either as monotherapy in selected patients or in combination with chemotherapy [22, 23]. However, the efficacy of anti-PD-(L)1 inhibitors against SCLC is limited [24]. In Japan, amrubicin is a commonly used treatment for relapsed SCLC, and is recommended in the Japanese Lung Cancer Society clinical practice guidelines [7]. Tarlatamab is an emerging immunotherapeutic agent with a mechanism of action distinct from that of immune checkpoint inhibitors [25]. In Japan, tarlatamab is included among treatment options in the subsequent-line setting for patients with advanced SCLC [14]. Despite recent advances, the treatment of SCLC remains challenging due to the limited durability of existing treatments and the frequent development of therapeutic resistance [26]. DeLLphi-304 was the first trial to demonstrate that tarlatamab, as a single agent, significantly improved OS compared to active treatment [15]. The present analysis of DeLLphi-304 shows that tarlatamab, a novel therapeutic agent in Japanese patients, numerically improved OS and DOR. These subpopulation results from the DeLLphi-304 trial, considered in the context of existing and emerging treatment options, support tarlatamab as a potential new standard of care for second-line treatment in Japanese patients with SCLC.

### Strengths and limitations

This exploratory analysis of Japanese patients from the DeLLphi-304 trial was not powered to detect statistically significant differences between treatment arms and was not a randomized comparison given that randomization related to tarlatamab versus standard of care chemotherapy. Information regarding management strategies was also not systematically collected in the study and, therefore, could not be evaluated. Another limitation relates to potential bias in that the improved efficacy with tarlatamab in this subpopulation analysis can at least partially be explained by baseline characteristics such as lower median age, the lower proportion of brain and liver metastases, and the higher proportion platinum-sensitive patients in the tarlatamab arm compared to the amrubicin arm. Nevertheless, the trends in efficacy were consistent with those in the global trial. Interpretation and generalization of the findings are also limited by the small sample size of Japanese patients in this analysis. Longer-term follow-up is warranted to further characterize the clinical benefit of tarlatamab in the Japanese subpopulation.

## Conclusions

In conclusion, tarlatamab demonstrated numerically longer OS and DOR, as well as a higher ORR but numerically shorter PFS versus amrubicin in the Japanese subpopulation with relapsed SCLC from the DeLLphi-304 trial. However, imbalance in several baseline characteristics, including chemotherapy-free interval and presence of brain metastases, together with a small sample size, are the limitations of this subgroup analysis. No new safety signals were identified with tarlatamab treatment. Consistent with findings in the global population, these results provide supportive evidence for the use of tarlatamab in Japanese patients with SCLC.

## Supplementary Information

Below is the link to the electronic supplementary material.

Supplementary Material 1

Supplementary Material 2

## Data Availability

Qualified researchers may request data from Amgen clinical studies. Further details are available at Amgen’s website on Clinical Trial Data Sharing Requests https://wwwext.amgen.com/science/clinical-trials/clinical-data-transparency-practices/clinical-trial-data-sharing-request
