# Supplementary material for: Tarlatamab in small cell lung cancer following platinum-based chemotherapy: subgroup analysis of Japanese patients from the DeLLphi-304 study
Source: Int J Clin Oncol. 2026 Sep 19;31(11):2784–93. doi: 10.1007/s10147-026-03192-y (PMC13710598; doi:10.1007/s10147-026-03192-y)
Supplement: Supplementary file 1 [file 10147_2026_3192_MOESM1_ESM.docx]

**Journal name:** International Journal of Clinical Oncology

**Title:** Tarlatamab in small cell lung cancer following platinum-based chemotherapy: subgroup analysis of Japanese patients from the DeLLphi-304 study

**Authors:** Takayasu Kurata,^1^ Shunichi Sugawara,^2^ Hiroaki Akamatsu,^3^ Kazushige Wakuda,^4^ Koichi Azuma,^5^ Takayuki Takahama,^6^ Hiroshi Kagamu,^7^ Hiroshi Yokouchi,^8^ Miyako Satouchi,^9^ Shuji Murakami,^10^ Kadoaki Ohashi,^11^ Makoto Nishio,^12^ Kazumi Nishino,^13^ Hiroki Izumi,^14^ Hiroshi Tanaka,^15^ Taichi Miyawaki,^16^ Shuang Huang,^17^ Upen Patil,^17^ Jihyun Park,^18^ Tatsuya Yoshida^19^

**Affiliations:** ^1^Department of Thoracic Oncology, Kansai Medical University Hospital, Osaka, Japan; ^2^Sendai Kousei Hospital, Miyagi, Japan; ^3^Internal Medicine III, Wakayama Medical University Hospital, Wakayama, Japan; ^4^Division of Thoracic Oncology, Shizuoka Cancer Center, Shizuoka, Japan; ^5^Division of Respirology, Neurology, and Rheumatology, Department of Internal Medicine, Kurume University School of Medicine, Fukuoka, Japan; ^6^Department of Medical Oncology, Kindai University Faculty of Medicine, Osaka-Sayama, Japan; ^7^Department of Respiratory Medicine, International Medical Center, Saitama Medical University, Saitama, Japan; ^8^Department of Respiratory Medicine, NHO Hokkaido Cancer Center, Sapporo, Japan; ^9^Department of Thoracic Oncology, Hyogo Cancer Center, Akashi, Japan; ^10^Department of Thoracic Oncology, Kanagawa Prefectural Hospital Organization Kanagawa Cancer Center, Yokohama, Japan; ^11^Department of Respiratory Medicine, Okayama University Hospital, Okayama, Japan; ^12^Department of Thoracic Medical Oncology, The Cancer Institute Hospital of Japanese Foundation for Cancer Research, Tokyo, Japan; ^13^Department of Thoracic Oncology, Osaka International Cancer Institute, Osaka, Japan; ^14^Department of Thoracic Oncology, National Cancer Center Hospital East, Chiba, Japan; ^15^Department of Internal Medicine, Niigata Cancer Center Hospital, Niigata, Japan; ^16^Department of Respiratory Medicine, Graduate School of Medicine, Juntendo University, Tokyo, Japan; ^17^Amgen Inc., Thousand Oaks, California, USA; ^18^Amgen K.K., Tokyo, Japan; ^19^Department of Thoracic Oncology, National Cancer Center Hospital, Tokyo, Japan.

**Corresponding Author:** Takayasu Kurata

Department of Thoracic Oncology, Kansai Medical University Hospital, 2-3-1 Shin-machi, Hirakata city, Osaka 573-1191, Japan

**E-mail:** [kurata.tak@kmu.ac.jp](mailto:kurata.tak@kmu.ac.jp)

**Supplementary Information**

**Supplementary Table 1** Inclusion and exclusion criteria

**Inclusion criteria**

Subjects are eligible to be included in the study only if all of the following criteria apply:

- Subject has provided informed consent prior to initiation of any study specific activities/procedures.
- Age ≥ 18 years (or legal adult age within country, whichever is older) at the time of signing the informed consent.
- Histologically or cytologically confirmed relapsed/refractory SCLC.
- Subject has progressed or recurred following 1 platinum-based regimen:
  - Documented first disease progression must be during or following first-line platinum-based systemic chemotherapy for ES or LS disease
  - Patients who received treatment for LS disease who recur are eligible
  - Patients who received adjuvant EP after resection of their SCLC who recur are eligible
  - In countries where SOC first line systemic treatment for ES disease includes platinum containing chemotherapy in combination with PD-(L)1 inhibitor, it is required that patients have failed PD-(L)1 inhibitor as part of their first line systemic treatment or are ineligible to receive PD-(L)1 inhibitor therapy
- Provision of evaluable tumor sample for central testing. Tumor sample must be either archival (FFPE blocks collected within 5 years, or slides sectioned within 30 days prior to receipt at the central lab), or a fresh core needle biopsy. Formalin-fixed, paraffin-embedded FNA/cytology samples are allowed (pleural effusion samples are not permitted).
- Measurable disease as defined per RECIST 1.1 within the 21-day screening period.
  - Screening scans performed as SOC and prior to informed consent, may be used to confirm subject eligibility if completed within the 21-day screening period, provided that informed consent for the use of these scans is obtained prior to any transfer of data.
- Eastern Cooperative Oncology Group (ECOG) PS of 0 or 1.
- Minimum life expectancy of 12 weeks.
- Adequate organ function, defined as follows:
  - Hematological function:
    - Absolute neutrophil count ≥ 1.5 x 10^9^ /L
    - Platelet count ≥ 100 x 10^9^ /L
    - Hemoglobin > 9 g/dL (90 g/L)
  - Coagulation function:
    - Prothrombin time (PT)/international normalized ratio (INR) and partial thromboplastin time (PTT) or activated partial thromboplastin time (APTT) ≤ 1.5 x institutional upper limit of normal (ULN) except for subjects on receiving anticoagulation, who must be on a stable dose of anticoagulant therapy for 6 weeks prior to enrollment.
  - Renal function:
    - Estimated glomerular filtration rate (eGFR) based on Modification of Diet in Renal Disease (MDRD) calculation > 30 mL/min/1.73 m^2^
  - Hepatic function:
    - Aspartate aminotransferase (AST) and alanine aminotransferase (ALT) < 3 x ULN (or < 5 x ULN for subjects with liver involvement)
    - Total bilirubin (TBL) < 1.5 x ULN (or < 2 x ULN for subjects with liver involvement)
  - Pulmonary function:
    - No clinically significant pleural effusion. Pleural effusion managed with indwelling pleural catheter (e.g, PleurX) are allowed.
    - Baseline oxygen saturation > 90% on room air
  - Cardiac function:
    - Cardiac ejection fraction ≥ 50%, no clinically significant pericardial effusion as determined by an echocardiogram (ECHO) or multigated acquisition (MUGA) scan, and no clinically significant electrocardiogram (ECG) findings

**Exclusion Criteria**

Subjects are excluded from the study if any of the following criteria apply:

**Disease Related**

- Untreated or symptomatic central nervous system (CNS) metastases:
  - Subjects with asymptomatic CNS metastases are eligible if clinically stable for at least 4 weeks and do not require intervention (including use of corticosteroids).
  - Subjects with treated brain metastases are eligible provided the following criteria are met:
    - Subject is asymptomatic from brain metastases
    - Whole brain radiation or surgery was completed at least 2 weeks prior to first dose of study treatment (stereotactic radiosurgery completed at least 7 days prior to first dose of study treatment)
    - Any CNS disease is clinically stable, subject is off steroids for CNS disease for at least 5 days (unless steroids are indicated for a reason unrelated to CNS disease), and subject is off or on stable doses of anti-epileptic drugs at least 14 days prior to first dose of study treatment
- Diagnosis or evidence of leptomeningeal disease.
- Prior history of immune checkpoint inhibitors resulting in:
  - Any severe or life-threatening immune-mediated adverse event
  - History of immune-mediated encephalitis or other immune-mediated CNS event (any grade)
  - Grade ≥ 2 immune-mediated recurrent pneumonitis
  - Infusion-related reactions leading to permanent discontinuation of immunotherapy agent

Exception: Subjects with a history of immune checkpoint inhibitor-induced endocrinopathy which is clinically stable on replacement therapy.

**Other Medical Conditions**

- Active autoimmune disease that has required systemic treatment (except replacement therapy) within the past 2 years or any other diseases requiring immunosuppressive therapy while on study.
- History of solid organ transplantation.
- History of other malignancy within the past 2 years, with the following exceptions:
  - Low-risk malignancy treated with curative intent and with no known active disease present for ≥ 1 year before enrollment and believed to be at low risk for recurrence per investigator discretion.
  - Adequately treated non-melanoma skin cancer or lentigo maligna without evidence of disease.
  - Adequately treated cervical carcinoma in situ without evidence of disease.
  - Adequately treated breast ductal carcinoma in situ without evidence of disease.
  - Prostatic intraepithelial neoplasia without evidence of prostate cancer.
  - Adequately treated urothelial papillary noninvasive carcinoma or carcinoma in situ.
- Myocardial infarction and/or symptomatic congestive heart failure (New York Heart Association > class II) within 12 months prior to first dose of study treatment.
- History of arterial thrombosis (e.g., stroke or transient ischemic attack) within 12 months prior to first dose of study treatment.
- Known human immunodeficiency virus (HIV) infection, hepatitis C infection (subjects with hepatitis C that achieve a sustained virologic response after antiviral therapy are allowed), or hepatitis B infection (subjects with hepatitis B surface antigen [HBsAg] or core antibody that achieve sustained virologic response with antiviral therapy are permitted with a requirement for regular monitoring for reactivation for the duration of treatment on the study).
- Receiving systemic corticosteroid therapy or any other form of immunosuppressive therapy within 7 days prior to first dose of study treatment:
  - Prophylactic dexamethasone required by the protocol and any anti-emetic therapies are allowed
  - Low-dose corticosteroids (prednisone ≤10mg per day or equivalent is permitted during the trial)
- Subject with symptoms and/or clinical signs and/or radiographic signs that indicate an acute and/or uncontrolled active systemic infection within 7 days prior to the first dose of study treatment.
  - Subject has known active infection requiring parenteral antibiotic treatment. Upon completion of parenteral antibiotics and resolution of symptoms, the subject may be considered eligible for the study from an infection standpoint.
  - Note: Simple urinary tract infection (UTI) and uncomplicated bacterial pharyngitis are permitted if responding to active treatment. Subjects requiring oral antibiotics who have been afebrile for > 24 hours, have no leukocytosis, nor clinical signs of infection are eligible. Screening for chronic infectious conditions is not required unless otherwise noted as exclusion criteria.
- Evidence of interstitial lung disease or active, non-infectious pneumonitis.

**Prior/Concomitant Therapy**

- Prior therapy with tarlatamab or any of the SOC chemotherapy included as part of this trial.
- Prior therapy with any selective inhibitor of the DLL3 pathway.
- Subject received more than one prior systemic therapy regimen for SCLC (EP/anti-PD-(L)1 therapy followed by anti-PD-(L)1 maintenance therapy is considered one regimen).
- Prior anti-cancer therapy within 21 days prior to first dose of study treatment.

Exceptions:

- Subjects who received conventional chemotherapy are eligible if at least 14 days have elapsed and if all treatment-related toxicity has been resolved to Grade ≤1, or to levels dictated in the eligibility criteria, before first dose of study treatment, with the exception of alopecia or toxicities considered irreversible (defined as having been present and stable for > 30 days) which are not otherwise described in the exclusion criteria.
- Prior palliative radiotherapy must have been completed at least 7 days before the first dose of study treatment.
- Receiving anti-cancer therapy such as chemotherapy, immunotherapy, or targeted therapy. Patients who are receiving adjuvant hormonal therapy for resected breast cancer may be eligible (refer also to exclusion related to history of other malignancies).
- Any herbal or prescription/non-prescription medications known to inhibit membrane transporters P-glycoprotein (P-gp) and/or breast cancer resistance protein (BCRP) (including but not limited to cyclosporine, clarithromycin, itraconazole, or ketoconazole) within 7 days prior to the first dose of study treatment.
- Any herbal or prescription/non-prescription medications known to be moderate or strong inhibitors of cytochrome P450 3A (CYP3A) enzymes (including but not limited to clarithromycin, itraconazole, ketoconazole) within 7 days prior to the first dose of study treatment.
- Any herbal or prescription/non-prescription medications known to be moderate or strong inducers of CYP3A enzymes (including but not limited to efavirenz, phenobarbital, phenytoin, rifampin, St John’s Wort) within 28 days prior to first dose of study treatment.
- Subjects who have reached the limit dose of prior treatment with cardiotoxic drugs such as other anthracyclines (the total dose of daunorubicin hydrochloride is 25 mg/kg body weight, the total dose of doxorubicin hydrochloride is 500 mg/m^2^ body surface area [BSA], the total dose of epirubicin hydrochloride is 900 mg/m^2^ BSA, the total dose of pirarubicin hydrochloride is 950 mg/m^2^ BSA, etc.)
- Major surgical procedures within 28 days prior to first dose of study treatment.
- Treatment with live virus, including live-attenuated vaccination, within 14 days prior to the first dose of study treatment. Inactive vaccines (e.g., non-live or non-replicating agent) and live viral non-replicating vaccines (e.g., Jynneos for Monkeypox infection) within 3 days prior to first dose of study treatment.

**Prior/Concurrent Clinical Study Experience**

- Currently receiving treatment in another investigational device or drug study, or less than 30 days since ending treatment on another investigational device or drug study(ies). Other investigational procedures while participating in this study are excluded.

**Diagnostic Assessments**

- Any previous diagnosis of transformed non-small cell lung cancer (NSCLC), epidermal growth factor receptor (EGFR) activating mutation positive NSCLC that has transformed to SCLC, or mixed SCLC NSCLC histology.

**Other Exclusions**

- Female subjects of childbearing potential unwilling to use protocol specified method of contraception during treatment and for an additional 72 days after the last dose of tarlatamab (Note: contraception requirements for SOC therapies are based on regional prescribing information).
- Female subjects who are breastfeeding or who plan to breastfeed while on study through 72 days after the last dose of tarlatamab (Note: breastfeeding restrictions for SOC therapies are based on regional prescribing information).
- Female subjects planning to become pregnant or donate eggs while on study through 72 days after the last dose of tarlatamab (Note: contraception requirements for SOC therapies are based on regional prescribing information).
- Female subjects of childbearing potential with a positive pregnancy test assessed at screening by a serum pregnancy test.
- Male subjects with a female partner of childbearing potential who are unwilling to practice sexual abstinence (refrain from heterosexual intercourse) or use contraception during treatment and for an additional 132 days after the last dose of tarlatamab.
- Male subjects with a pregnant partner who are unwilling to practice abstinence or use a condom during treatment and for an additional 132 days after the last dose of tarlatamab.
- Male subjects unwilling to abstain from donating sperm during treatment and for an additional 132 days after the last dose of tarlatamab.
- Subject has known sensitivity or is contraindicated to any of the products or components to be administered during dosing.
- Subject likely to not be available to complete all protocol-required study visits or procedures, and/or to comply with all required study procedures (e.g., Clinical Outcome Assessments) to the best of the subject and investigator’s knowledge.
- History or evidence of any other clinically significant disorder, condition or disease (with the exception of those outlined above) that, in the opinion of the investigator or Amgen Medical Monitor, if consulted, would pose a risk to subject safety or interfere with the study evaluation, procedures, or completion.

**Supplementary Table 2** Incidence of treatment-related adverse events by preferred term in the Japanese subpopulation (Safety analysis set)

| **Preferred Term** | **Tarlatamab (n = 12)** | **Amrubicin (n = 23)** |
| --- | --- | --- |
| Number of subjects reporting treatment-related adverse events | 12 (100.0) | 21 (91.3) |
| Cytokine release syndrome | 9 (75.0) | 0 (0.0) |
| Dysgeusia | 8 (66.7) | 1 (4.3) |
| Anemia | 2 (16.7) | 12 (52.2) |
| Neutropenia | 2 (16.7) | 8 (34.8) |
| Constipation | 2 (16.7) | 6 (26.1) |
| Leukopenia | 1 (8.3) | 6 (26.1) |
| Decreased appetite | 1 (8.3) | 4 (17.4) |
| Stomatitis | 1 (8.3) | 3 (13.0) |
| Pyrexia | 1 (8.3) | 2 (8.7) |
| Alanine aminotransferase increased | 1 (8.3) | 1 (4.3) |
| Aspartate aminotransferase increased | 1 (8.3) | 0 (0.0) |
| Dermatitis | 1 (8.3) | 0 (0.0) |
| Dry skin | 1 (8.3) | 0 (0.0) |
| Hypoesthesia | 1 (8.3) | 0 (0.0) |
| Hypozincemia | 1 (8.3) | 0 (0.0) |
| Immune effector cell-associated neurotoxicity syndrome | 1 (8.3) | 0 (0.0) |
| Lymphopenia | 1 (8.3) | 0 (0.0) |
| Myalgia | 1 (8.3) | 0 (0.0) |
| Venous thrombosis limb | 1 (8.3) | 0 (0.0) |
| Weight decreased | 1 (8.3) | 0 (0.0) |
| Febrile neutropenia | 0 (0.0) | 7 (30.4) |
| Malaise | 0 (0.0) | 6 (26.1) |
| Thrombocytopenia | 0 (0.0) | 5 (21.7) |
| Nausea | 0 (0.0) | 4 (17.4) |
| Platelet count decreased | 0 (0.0) | 3 (13.0) |
| Alopecia | 0 (0.0) | 2 (8.7) |
| Diarrhea | 0 (0.0) | 2 (8.7) |
| Neutrophil count decreased | 0 (0.0) | 2 (8.7) |
| Phlebitis | 0 (0.0) | 2 (8.7) |
| Pneumonia | 0 (0.0) | 2 (8.7) |
| White blood cell count decreased | 0 (0.0) | 2 (8.7) |
| Amenorrhea | 0 (0.0) | 1 (4.3) |
| Cough | 0 (0.0) | 1 (4.3) |
| Eczema | 0 (0.0) | 1 (4.3) |
| Fatigue | 0 (0.0) | 1 (4.3) |
| Hiccups | 0 (0.0) | 1 (4.3) |
| Hypoalbuminemia | 0 (0.0) | 1 (4.3) |
| Infectious pleural effusion | 0 (0.0) | 1 (4.3) |
| Infusion related reaction | 0 (0.0) | 1 (4.3) |
| Infusion site extravasation | 0 (0.0) | 1 (4.3) |
| Myasthenic syndrome | 0 (0.0) | 1 (4.3) |
| Taste disorder | 0 (0.0) | 1 (4.3) |
| Vasculitis | 0 (0.0) | 1 (4.3) |
| Vomiting | 0 (0.0) | 1 (4.3) |

Note: Treatment-emergent adverse events (TEAEs) were adverse events that occurred or worsened after initiation of study treatment, regardless of causality. Treatment-related adverse events (TRAEs) were TEAEs considered by the investigator to have a reasonable possibility of being related to study treatment.

**Supplementary Table 3.** Incidence of treatment-emergent adverse events and treatment-related adverse events of Grade ≥ 3 by system organ class and preferred term in the Japanese subpopulation (Safety analysis set)

|  | **Treatment-emergent adverse events** | | **Treatment-related adverse events** | |
| --- | --- | --- | --- | --- |
| **System organ class**  **Preferred term** | **Tarlatamab (n = 12)** | **Amrubicin (n = 23)** | **Tarlatamab (n = 12)** | **Amrubicin (n = 23)** |
| Number of subjects reporting Grade ≥ 3 events | 8 (66.7) | 17 (73.9) | 1 (8.3) | 13 (56.5) |
| Blood and lymphatic system disorders | 5 (41.7) | 14 (60.9) | 1 (8.3) | 12 (52.2) |
| Neutropenia | 3 (25.0) | 9 (39.1) | 1 (8.3) | 7 (30.4) |
| Febrile neutropenia | 2 (16.7) | 7 (30.4) | 0 (0.0) | 7 (30.4) |
| Leukopenia | 1 (8.3) | 6 (26.1) | 0 (0.0) | 6 (26.1) |
| Anemia | 0 (0.0) | 6 (26.1) | 0 (0.0) | 6 (26.1) |
| Cardiac disorders | 1 (8.3) | 1 (4.3) | 0 (0.0) | 0 (0.0) |
| Sinus node dysfunction | 1 (8.3) | 0 (0.0) | 0 (0.0) | 0 (0.0) |
| Cardiac failure | 0 (0.0) | 1 (4.3) | 0 (0.0) | 0 (0.0) |
| Infections and infestations | 1 (8.3) | 5 (21.7) | 0 (0.0) | 3 (13.0) |
| Pneumonia | 1 (8.3) | 3 (13.0) | 0 (0.0) | 2 (8.7) |
| Infectious pleural effusion | 0 (0.0) | 1 (4.3) | 0 (0.0) | 1 (4.3) |
| Pneumonia aspiration | 0 (0.0) | 1 (4.3) | 0 (0.0) | 0 (0.0) |
| Investigations | 2 (16.7) | 4 (17.4) | 0 (0.0) | 3 (13.0) |
| Neutrophil count decreased | 2 (16.7) | 3 (13.0) | 0 (0.0) | 2 (8.7) |
| White blood cell count decreased | 1 (8.3) | 2 (8.7) | 0 (0.0) | 1 (4.3) |
| Amylase increased | 1 (8.3) | 0 (0.0) | 0 (0.0) | 0 (0.0) |
| Lipase increased | 1 (8.3) | 0 (0.0) | 0 (0.0) | 0 (0.0) |
| Platelet count decreased | 0 (0.0) | 1 (4.3) | 0 (0.0) | 1 (4.3) |
| Weight decreased | 0 (0.0) | 1 (4.3) | 0 (0.0) | 0 (0.0) |
| Metabolism and nutrition disorders | 1 (8.3) | 5 (21.7) | 0 (0.0) | 3 (13.0) |
| Decreased appetite | 1 (8.3) | 2 (8.7) | 0 (0.0) | 2 (8.7) |
| Hypoalbuminemia | 0 (0.0) | 1 (4.3) | 0 (0.0) | 1 (4.3) |
| Hypokalemia | 0 (0.0) | 1 (4.3) | 0 (0.0) | 0 (0.0) |
| Hyponatremia | 0 (0.0) | 1 (4.3) | 0 (0.0) | 0 (0.0) |
| Neoplasms benign, malignant and unspecified (incl cysts and polyps) | 0 (0.0) | 1 (4.3) | 0 (0.0) | 0 (0.0) |
| Lymphangiosis carcinomatosa | 0 (0.0) | 1 (4.3) | 0 (0.0) | 0 (0.0) |
| Nervous system disorders | 0 (0.0) | 2 (8.7) | 0 (0.0) | 0 (0.0) |
| Spinal cord compression | 0 (0.0) | 1 (4.3) | 0 (0.0) | 0 (0.0) |
| Syncope | 0 (0.0) | 1 (4.3) | 0 (0.0) | 0 (0.0) |

Note: Treatment-emergent adverse events (TEAEs) were adverse events that occurred or worsened after initiation of study treatment, regardless of causality. Treatment-related adverse events (TRAEs) were TEAEs considered by the investigator to have a reasonable possibility of being related to study treatment.
